# Genetic Association of Interleukin-31 Gene Polymorphisms with Epithelial Ovarian Cancer in Chinese Population

**DOI:** 10.1155/2018/3503858

**Published:** 2018-02-01

**Authors:** Chenlu Liu, Yanyun Wang, Huizi Song, Qin Li, Yan Zhang, Peng Chen, Yaping Song, Min Su, Qin Huang, Mei Wang, Bin Zhou, Lin Zhang

**Affiliations:** ^1^Laboratory of Molecular and Translational Medicine, Key Laboratory of Birth Defects and Related Diseases of Women and Children of Ministry of Education, West China Second University Hospital, Sichuan University, Chengdu, Sichuan 610061, China; ^2^Department of Cardiology, West China Hospital of Sichuan University, Chengdu, Sichuan 610041, China; ^3^Department of Immunology, West China School of Preclinical and Forensic Medicine, Sichuan University, Chengdu, Sichuan 610041, China; ^4^Department of Pathology, West China Second University Hospital, Sichuan University, Chengdu, Sichuan 610061, China; ^5^Department of Forensic Medicine, Nanjing Medical University, Nanjing 210029, China

## Abstract

Roles of interleukin-31 (IL-31) in the development and progression of human epithelial ovarian cancer are largely unknown. Studies report that the polymorphisms, rs7977932 C>G and rs4758680 C>A in *IL-31*, affect the expression level of IL-31. In the present study, we examined 412 patients with epithelial ovarian cancer and 428 healthy individuals to explore whether these polymorphisms are associated with the epithelial ovarian cancer in Chinese women. The genotype of the polymorphisms in each individual was identified. The associations of the polymorphisms with patients' clinical characteristics and outcomes were evaluated. For rs7977932, the frequency of the CG/GG was significantly decreased in patients with epithelial ovarian cancer. However, the frequency of the rs4758680 CA/AA was significantly increased in those patients. Moreover, the frequency of rs7977932 CG/GG genotype was significantly higher in patients with less advanced FIGO stages. Kaplan-Meier curve showed that patients with CG/GG genotypes of rs7977932 had a decreased risk for recurrence compared to those with CC genotype. Our findings suggested that rs7977932 and rs4758680 of *IL-31* may be associated with the development and progression of the epithelial ovarian cancer in the Chinese population. IL-31, therefore, may be a potential therapeutic target for the development of drugs to treat the disease.

## 1. Introduction

Ovarian cancer is the most lethal gynecological cancer, and the overall five-year survival rate is less than 40% [1]. The pathogenesis of ovarian cancer, especially the epithelial ovarian cancer, is still largely unknown. Data from several investigations indicate that mutations in genes, such as tumor protein 53, breast cancers 1 and 2, are associated with the development of epithelial ovarian cancer [2–4]. In addition, other factors, such as inflammatory cytokines, may also be implicated in the development of epithelial ovarian cancer at all stages [1].

Interleukin-31 (IL-31) is mainly produced by the activated T helper type 2 cells as well as other types of cells, including monocytes, macrophages, and immature and mature dendritic and mast cells [5–7]. It has been reported that IL-31 may be involved in the pathogenesis of cutaneous allergic diseases and atopic dermatitis [8]. Recent studies suggest that IL-31 may also contribute to hematopoietic malignancies and tumor growth in human lymphoma [5, 9–11]. Results from other investigations demonstrate that IL-31 can significantly increase expressions of epidermal growth factor and vascular endothelial growth factor [12, 13]. Thus, IL-31 has been considered an angiogenic factor to lung epithelial cells, which may play a role in the development of lung cancer [12, 14]. In contrast to the aforementioned finds, the results from other independent investigations demonstrate that IL-31 is highly effective in suppressing the proliferation of lung epithelial cells and has the antiproliferative effect on the cells [14, 15]. IL-31 and its receptor, IL31RA, are highly expressed in tumor specimens from ovarian cancer patients and other human and mouse cancer cell lines [16]. IL-31 may have anticancer effects on certain kinds of tumors. For instance, murine colon carcinoma cells without IL-31 have greater invasive, migratory, and tumor growth properties, while these effects can be reversed by supplementing exogenous IL-31 to the cells. Moreover, the tumor growth in mice can also be inhibited by the administration of exogenous IL-31 [16]. The aforementioned information prompted us to investigate potential roles of IL-31 in epithelial ovarian cancer.

Single nucleotide polymorphisms (SNPs) in *IL-31* provide us with an opportunity to investigate potential effects of the factor in the development and progression of epithelial ovarian cancer in humans. The SNP is the most common type of the genetic variation in human genome. A SNP can alter the expression of a gene, which leads to a specific phenotype of cells. For example, polymorphisms in IL-31 have been associated with the development of diseases and cancer [16–20]. In particular, systemic lupus erythematosus (SLE) patients carrying rs7977932 G allele in *IL-31* have the high-level protein of IL-31 in serum [19]. Recent data show that the level of IL-31 mRNA in white blood cells is increased in dilated cardiomyopathy (DCM) patients, associated with a lower frequency of the CA/AA at rs4758680, in *IL-31* [20]. However, there is no information of the polymorphisms, rs7977932 and rs4758680 of *IL-31*, in epithelial ovarian cancer patients.

In this present investigation, we retrospectively examined the genotypes of the rs7977932 and rs4758680 in 412 epithelial ovarian cancer patients. The rs7977932 and rs4758680 locate within *IL-31* and are 2031 base pairs apart. The associations of the rs7977932 and rs4758680, with the epithelial ovarian cancer, were statistically calculated. The associations of the rs7977932 and rs4758680 with the cancer features, survival outcome, and recurrence were also evaluated.

## 2. Materials and Methods

### 2.1. Study Subjects

Four hundred and twelve patients, aged 51.2 years (average) ± 9.6 years (SD), were diagnosed with epithelial ovarian cancer and treated at the West China Second University Hospital. Four hundred and twenty-eight healthy female individuals, aged 49.7 years (average) ± 13.5 years (SD), were enrolled in this hospital-based retrospective case-control study (Table 1). We excluded individuals with the borderline ovarian tumors, two or more different malignancies and autoimmune diseases from this study. The tumor stage was classified based on the International Federation of Gynecology and Obstetrics ovarian cancer staging criteria (FIGO, 2014). Healthy individuals were from a routine physical examination in the hospital. They had no personal or family history of cancer, autoimmune diseases, or other serious illnesses. The present investigation has been approved by the Ethics Committee of the West China Second Hospital, and the consent form was obtained from all participants.

### 2.2. Determination of Genotypes

The SNPs, rs7977932 (C>G) and rs4758680 (C>A), were genotyped by restriction fragment length polymorphism (RFLP) of PCR products. The three kinds of SNP genotypes were also confirmed by direct DNA sequence. For PCR, the genomic DNA of each patient was isolated from the paraffin section of the tumor tissue sample by the DNA isolation kit (BioTeke, Beijing, China). Genomic DNAs of the healthy individuals were extracted from 200 *μ*l EDTA-anticoagulated peripheral blood sample by the whole blood DNA isolation kit (BioTeke, Beijing, China). For genotyping of the rs7977932, the primers for PCR were forward 5′-GGTCAGTGTTGGGTTTGCAATG-3′ and reverse 5′-TTGGTGATGGCACAGCCTCATA-3′. For the rs4758680, the primers for PCR were forward 5′-AGGTCTGTGGGTGGAGACAG-3′ and reverse 5′-TTTCCCCCGAGATAAGATGA-3′. The PCR reaction was 25 *μ*l including 0.5 mmol/L each PCR primer, 100 ng of the genomic DNA, 0.15 mmol/L of dNTPs, 2.5 *μ*l of 10 × Taq Buffer, and 1 U of Taq DNA polymerase (BioTeke, Beijing, China). The PCR condition was 95°C for 4 minutes followed by 36 cycles at 95°C for 30 seconds, 60°C for 30 seconds, and 72°C for 30 seconds and a final extension by 72°C for 10 minutes. PCR products were then digested by the restriction enzyme ScrFI (for rs7977932) overnight and the enzyme MboII (for rs4758680) for 35 minutes at 37°C. The rs7977932 allele C was not digested by ScrFI, and the size of the PCR product was 131 base pairs. The rs7977932 allele G was digested by ScrFI to generate two DNA fragments, 74 and 57 base pairs. The rs4758680 allele A was not digested by MboII, and the size of the PCR product was 130 base pairs. The rs4758680 allele C was digested by MboII to generate two DNA fragments, 100 and 30 base pairs. RFLP was performed by the use of 6% polyacrylamide gels with silver staining. Five percent of the DNA samples were randomly selected and reanalyzed by RFLP to confirm the result. Automatic DNA sequencing was used to verify the three genotypes of the rs7977932 and rs4758680. DNA template was prepared, and cycle sequencing reaction was conducted by Sanger's dideoxy terminator method. After the postsequencing reaction cleanup, fragments were separated by capillary electrophoresis on ABI 96-capillary 3730XL sequencer.

### 2.3. Patients' Clinical Characteristics and Follow-Up

The information of tumor stage (FIGO stages I + II and III + IV), tumor grade (G1 + G2 and grade G3), the histological type (serous and others), and ages (<50 and ≥50 years old) was collected from the medical records of the 412 patients. Based on the records, 167 epithelial ovarian cancer patients were randomly recruited in the follow-up study for the survival outcomes and tumor recurrence analysis. We collected the patient clinical outcomes from the primary date of the diagnosis to the cancer recurrence or death. The clinical follow-up study was performed in a blind manner with regard to patient status.

### 2.4. Statistical Analysis

The differences of genotypes between the epithelial ovarian cancer patients and the controls were analyzed using SNPStats online software (https://www.snpstats.net/snpstats/start.htm), which provided the genotypic association including codominant, dominant, recessive, or overdominant genetic models. Odd ratios (OR) with a 95% confidence interval (95%CI) were obtained accordingly. Hardy-Weinberg equilibrium and the association of the genotypes and the alleles with patients' clinical characteristics were assessed by chi-square analysis. Kaplan-Meier curve and Cox proportional hazard models were applied for the evaluation of the role of IL-31 SNPs and other factors on prognosis of epithelial ovarian cancer patients. The level of the statistical significance was set at *P* value < 0.05.

## 3. Results

### 3.1. The SNPs of IL-31, rs7977932, and rs4758680, in Epithelial Ovarian Cancer Patients

The SNPs rs7977932 and rs4758680 were genotyped in the 412 patients with epithelial ovarian cancer and 428 control individuals. Genotypes of the SNPs, rs7977932 (CC, CG, and GG) and rs4758680 (CC, CA, and AA), were identified. The frequencies of all observed genotypes in both patients and controls were in agreement with the Hardy-Weinberg equilibrium (*P* > 0.05). Genotype distributions and allele frequencies of the SNPs of the *IL-31* in patients and controls are shown in Table 2. For the rs7977932 C>G polymorphism, G allele carriers were associated with a significantly decreased epithelial ovarian cancer development (*P* = 0.0024, OR = 0.55, 95% CI = 0.37–0.81) in a dominant model (CG/GG versus CC). Similarly, significant associations were also observed in a codominant model (CG versus CC; *P* = 0.01, OR = 0.54, 95% CI = 0.36–0.82) and an overdominant model (CG versus CC/GG; *P* = 0.0033, OR = 0.55, 95% CI = 0.36–0.83). And the overall allele G is significantly lower in epithelial ovarian cancer patients than in the controls (G versus C; *P* = 0.0033, OR = 0.58, 95% CI = 0.40–0.84). For the rs4758680 C>A polymorphism, A allele carriers were associated with a significantly increased epithelial ovarian cancer development (*P* = 0.012, OR = 1.45, 95% CI = 1.09–1.92) in a dominant model (CA/AA versus CC). Similarly, significant associations were also observed in a codominant model (CA versus CC; *P* = 0.042, OR = 1.45, 95%CI = 1.09–1.96) and an overdominant model (CA versus CC/AA; *P* = 0.017, OR = 1.43, 95%CI = 1.06–1.92). And the overall allele A is significantly higher in epithelial ovarian cancer patients than the controls (A versus C; *P* = 0.024, OR = 1.32, 95% CI = 1.04–1.67).

### 3.2. IL-31 SNPs and Epithelial Ovarian Cancer Patients' Characteristics

To further evaluate whether the genotype and allele polymorphism were associated with certain clinical features of epithelial ovarian cancer patients, we performed the stratification analyses for genotype and allele distribution in epithelial ovarian cancer patients with different FIGO stages (I + II and III + IV), tumor grades (G1 + G2 and grade G3), histological types (serous and others), and ages (<50 and ≥50 years old). No statistically significant differences were found for any subgroups of the two SNPs except for the FIGO stages. For rs7977932, the percentage of CG/GG genotype was significantly higher in patients at early stages (I and II) compared with those at advanced stages (III and IV) (*P* = 0.042, OR = 0.50, 95% CI = 0.26–0.99). The proportion of patients with G allele was also significantly higher at early stages than at advanced stages (*P* = 0.017, OR = 0.48, 95% CI = 0.26–0.88) (Table 3).

### 3.3. Survival and Recurrence Analysis

**Figure 1:**
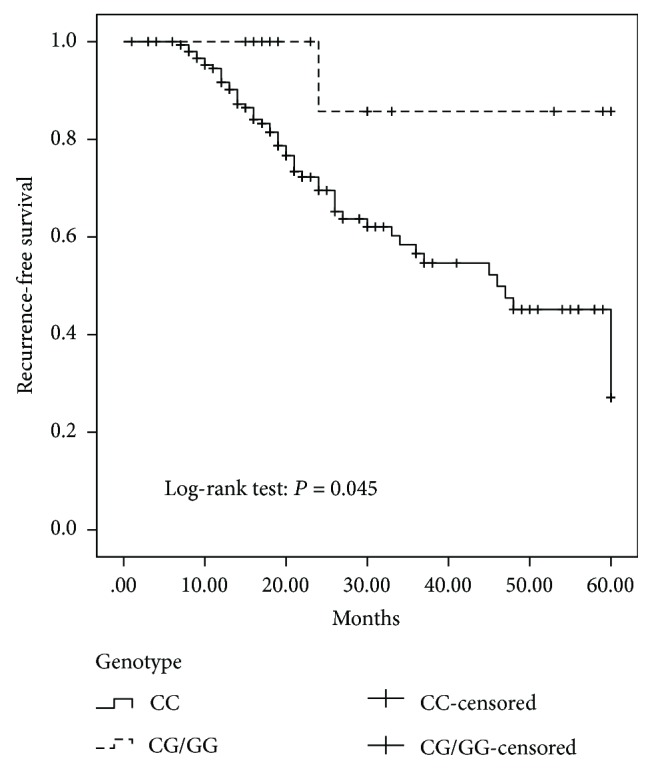
Kaplan-Meier recurrence-free survival curves of the epithelial ovarian cancer patients. For rs7977932, there is a significant difference between the CC and CG/GG, *P* = 0.045.

In the present study, no significant associations between the two SNPs (rs7977932 and rs4758680) and the overall survival of the epithelial ovarian cancer patients were observed by both the univariate and multivariate analysis (data not shown). However, the univariate analysis (Kaplan-Meier curve) showed that patients with CG/GG genotypes of rs7977932 had a decreased risk for recurrence compared to those with CC genotype (*P* = 0.045, Figure 1). No significant association was found between rs4758680 and the recurrence outcomes of the patients. Then, multivariate analysis using Cox proportional hazard models, adjusted by FIGO stage, histology type, and tumor grade, was carried out to examine the effects of *IL-31* polymorphisms on patients' outcomes. *IL-31* polymorphisms rs7977932 yielded no independent prognostic effect (*P* = 0.199), while FIGO stage (HR = 0.249, 95% CI = 0.074–0.837, *P* = 0.025) and histological types (HR = 2.207, 95% CI = 1.021–4.774, *P* = 0.044) were independent prognostic factors for recurrence outcomes.

## 4. Discussion

In this investigation, the frequency of the CG/GG of the rs7977932 was significantly decreased in the patients than in the healthy individuals. This suggested that the lower frequency of the CG/GG increased the risk of the epithelial ovarian cancer. It indicates that the CG/GG may be associated with the protective effect of IL-31 against the development of the epithelial ovarian cancer. Our result is in line with the previous observations that the CG polymorphism is associated with the elevated level of IL-31 in serum [16]. The increase in the expression of IL-31 may be able to reverse cancer invasive and migratory abilities and inhibit tumor growth [19]. For the rs4758680, the frequency of the CA/AA was significantly higher in the epithelial ovarian cancer patients than in the healthy individuals. This suggested that the CA/AA might be a risk factor for the development of the epithelial ovarian cancer. A recent study reports that the level of IL-31 mRNA expressed in white blood cells is increased in DCM compared with that in healthy individuals, while the frequency of the CA/AA genotypes is lower in DCM [20]. Although the genotypes of CA/AA may affect the expression of *IL-31*, there is no direct evidence to show whether the higher CA/AA is associated with the reduced level of IL-31. Taken all these findings together, it indicates that the CG/GG associated with the increased expression of IL-31 may have an antiepithelial ovarian cancer effect in a Chinese population.

We also found that the percentage of the CG/GG was significantly higher in patients with the epithelial ovarian cancer at the early stages I and II, compared with that in patients at the advanced stages III and IV. This suggests that the lower frequency of the CG/GG may lead to the development of more severe epithelial ovarian cancer. Thus, the increased level of IL-31 by the genotypes of the CG/GG may contribute to the reduction of the epithelial ovarian cancer severity. Our result is also in an agreement with the previous finding by others that IL31 is highly effective in suppressing the proliferation of lung epithelial cells [15].

In the follow-up investigation, we found that patients with CG/GG genotypes of rs7977932 had a decreased risk for recurrence compared to those with CC genotype. However, these genotypes were not independent risk factors for the prognosis of the patients with the epithelial ovarian cancer. Results from the Cox proportional hazard modeling showed that the independent, significant prognostic factors were FIGO stage and histological type. Variables, such as tumor grade, and the genotypes of the two SNPs (rs7977932 and rs4758680) were insignificant in predicting the epithelial ovarian cancer 5-year recurrence. These results partially support the finding by others that IL-31 might play an antiangiogenic role in tumor formation [16]. A lower recurrence risk of the epithelial ovarian cancer in patients with the CG/GG genotypes may represent the potential antiangiogenic effect of IL-31 since tumor growth is highly dependent on angiogenesis [21–23]. However, the FIGO stage and histological type play more dominant roles in the prognostic outcome of epithelial ovarian cancer, and these SNPs are not an independent prognostic factor for epithelial ovarian cancer. Interestingly, data from a study of patients with breast cancer demonstrate that there is a significant extended survival to breast cancer patients with a higher level expression of IL31RA [16, 24].

Lastly, in the present study, there were no associations between rs4758680 polymorphism and the patients' clinical characteristics such as epithelial ovarian cancer stages by FIGO, or between the rs4758680 polymorphism and the recurrence and survival risks of epithelial ovarian cancer. One of the possible explanations is that the polymorphism rs4758680 alone may not be sufficient enough to contribute to the stages and outcomes of the cancer.

## 5. Conclusion

Results from our present investigation indicate the significant association of the SNPs rs7977932 and rs4758680 in IL-31 with the development of epithelial ovarian cancer in a Chinese population. Combined with the findings from other independent research groups, it suggests that IL-31 can protect ovarian epithelial cells from being malignant. Thus, IL-31 may potentially be used as an antiovarian cancer drug to treat the disease [21].

## Figures and Tables

**Table 1:** Descriptive characteristics of the epithelial ovarian cancer patients.

Variable	Value
Sample size	*n* = 412
Mean age ± SD, range (years)	51.2 ± 9.6, 15–85
FIGO stage, number (%)	
I	43 (10.44%)
II	48 (11.65%)
III	297 (72.09%)
IV	24 (5.83%)
Tumor grade, number (%)	
G1	7 (1.70%)
G2	25 (6.07%)
G3	380 (92.23%)
Histological type, number (%)	
Serous	333 (80.83%)
Clear cell	16 (3.88%)
Endometrioid	9 (2.18%)
Mucinous	9 (2.18%)
Mixed and other	45 (10.92%)

**Table 2:** Distribution of SNPs in *IL-31* among the cases and controls and their association with the epithelial ovarian cancer.

		rs7977932					rs4758680			
		Case, *n* (%)	Control, *n* (%)	OR (95% CI)	*P* value		Case, *n* (%)	Control, *n* (%)	OR (95% CI)	*P* value
Model	Genotype					Genotype				
Codominant	CC	368 (89.3%)	351 (82%)	1.00		CC	252 (61.2%)	297 (69.4%)	1.00	
	CG	41 (9.9%)	72 (16.8%)	0.54 (0.36–0.82)	0.01^∗^	CA	141 (34.2%)	114 (26.6%)	1.45 (1.09–1.96)	0.042^∗^
	GG	3 (0.7%)	5 (1.2%)	0.57 (0.14–2.44)		AA	19 (4.6%)	17 (4%)	1.32 (0.67–2.56)	
Dominant	CC	368 (89.3%)	351 (82%)	1.00		CC	252 (61.2%)	297 (69.4%)	1.00	
	CG/GG	44 (10.7%)	77 (18%)	0.55 (0.37–0.81)	0.0024^∗^	CA/AA	160 (38.8%)	131 (30.6%)	1.45 (1.09–1.92)	0.012^∗^
Recessive	CC/CG	409 (99.3%)	423 (98.8%)	1.00		CC/CA	393 (95.4%)	411 (96%)	1.00	
	GG	3 (0.7%)	5 (1.2%)	0.62 (0.15–2.63)	0.51	AA	19 (4.6%)	17 (4%)	1.16 (0.60–2.27)	0.65
Overdominant	CC/GG	371 (90%)	356 (83.2%)	1.00		CC/AA	271 (65.8%)	314 (73.4%)	1.00	
	CG	41 (9.9%)	72 (16.8%)	0.55 (0.36–0.83)	0.0033^∗^	CA	141 (34.2%)	114 (26.6%)	1.43 (1.06–1.92)	0.017^∗^
	Allele					Allele				
	C	775 (94%)	774 (90%)	1.00		C	645 (78%)	708 (83%)	1.00	
	G	47 (6%)	82 (10%)	0.58 (0.40–0.84)	0.0033^∗^	A	179 (22%)	148 (17%)	1.32 (1.04–1.67)	0.024^∗^

*n* corresponds to the number of individuals; ^∗^*P* < 0.05.

**Table 3:** Association between SNPs, rs7977932, and rs4758680 and clinic features of the epithelial ovarian cancer.

Clinical features	rs7977932				Clinical features	rs4758680			
	Genotype frequency (%)		OR (95% CI)	*P* value		Genotype frequency (%)		OR (95% CI)	*P* value
FIGO stage	I-II	III-IV				I-II	III-IV		
CC	76 (83.52%)	292 (90.97%)	1.00		CC	55 (60.44%)	197 (61.37%)	1.00	
CG/GG	15 (16.48%)	29 (9.03%)	0.50 (0.26–0.99)	0.042^∗^	CA/AA	36 (39.56%)	124 (38.63%)	0.96 (0.60–1.55)	0.872
C	165 (90.66%)	612 (95.33%)	1.00		C	141 (77.47%)	504 (78.50%)	1.00	
G	17 (9.34%)	30 (4.67%)	0.48 (0.26–0.88)	0.017^∗^	A	41 (22.53%)	138 (21.50%)	0.94 (0.63–1.40)	0.766
Tumor grade	G1-G2	G3				G1-G2	G3		
CC	28 (87.50%)	340 (89.47%)	1.00		CC	21 (65.63%)	231 (60.79%)	1.00	
CG/GG	4 (12.50%)	40 (10.53%)	0.82 (0.28–2.47)	0.961	CA/AA	11 (34.38%)	149 (39.21%)	1.23 (0.58–2.63)	0.590
C	60 (93.75%)	717 (94.34%)	1.00		C	53 (82.81%)	592 (77.89%)	1.00	
G	4 (6.25%)	43 (5.66%)	0.90 (0.31–2.50)	0.779	A	11 (17.19%)	168 (22.11%)	1.37 (0.70–2.68)	0.360
Histological type	Serous	Others				Serous	Others		
CC	295 (88.59%)	73 (92.41%)	1.00		CC	202 (60.66%)	50 (63.29%)	1.00	
CG/GG	38 (11.41%)	6 (7.59%)	0.64 (0.26–1.57)	0.323	CA/AA	131 (39.34%)	29 (36.71%)	0.89 (0.54–1.49)	0.666
C	625 (93.84%)	152 (96.20%)	1.00		C	520 (78.08%)	125 (79.11%)	1.00	
G	41 (6.16%)	6 (3.80%)	0.60 (0.25–1.44)	0.250	A	146 (21.92%)	33 (20.89%)	0.94 (0.62–1.44)	0.777
Age (years)	<50	≥50				<50	≥50		
CC	164 (87.70%)	204 (90.67%)	1		CC	112 (59.89%)	140 (62.22%)	1	
CG/GG	23 (12.30%)	21 (9.33%)	0.73 (0.40–1.37)	0.332	CA/AA	75 (40.11%)	85 (37.78%)	0.91 (0.61–1.35)	0.629
C	349 (93.32%)	428 (95.11%)	1.00		C	288 (77.01%)	357 (79.33%)	1.00	
G	25 (6.68%)	22 (4.89%)	0.72 (0.40–1.30)	0.269	A	86 (22.99%)	93 (20.67%)	0.87 (0.63–1.22)	0.420

^∗^ *P* < 0.05.
